# Characterizing Cerebral Perfusion Changes in Subjective Cognitive Decline Using Single Photon Emission Computed Tomography: A Case-Control Study

**DOI:** 10.3390/jcm13226855

**Published:** 2024-11-14

**Authors:** Yu-Kai Lin, Li-Fan Lin, Chun-Hao Kao, Ing-Jou Chen, Cheng-Yi Cheng, Chia-Lin Tsai, Jiunn-Tay Lee, Yueh-Feng Sung, Chung-Hsing Chou, Shang-Yi Yen, Chuang-Hsin Chiu, Fu-Chi Yang

**Affiliations:** 1 Department of Neurology, Tri-Service General Hospital, National Defense Medical Center, Taipei 11490, Taiwan; yukai0907@ndmctsgh.edu.tw (Y.-K.L.);; 2 Graduate Institute of Medical Sciences, National Defense Medical Center, Taipei 11490, Taiwan; 3 Department of Nuclear Medicine, Tri-Service General Hospital, National Defense Medical Center, Taipei 11490, Taiwan

**Keywords:** regional cerebral blood flow, subjective cognitive decline, single photon emission computerized tomography, cognition, biomarkers

## Abstract

**Background/Objectives:** Subjective cognitive decline (SCD) may serve as an early indicator of Alzheimer’s disease (AD). This study investigates regional cerebral blood flow (rCBF) alterations in individuals with SCD using single photon emission computed tomography (SPECT). To characterize rCBF patterns in SCD patients compared to healthy controls and examine the relationship between rCBF and cognitive function. **Methods:** We compared rCBF in 20 SCD patients and 20 age- and sex-matched healthy controls using 99mTc-ECD SPECT imaging. Cognitive function was assessed using the Mini-Mental State Examination (MMSE), Clinical Dementia Rating (CDR), Geriatric Depression Scale (GDS), and Cognitive Abilities Screening Instrument (CASI). **Results:** SCD patients demonstrated significantly reduced rCBF in the right superior temporal gyrus (rSTG) (mean uptake ratio [UR] = 0.864 ± 0.090 vs. 1.030 ± 0.074, *p* < 0.001) and right caudate (mean UR = 0.783 ± 0.068 vs. 0.947 ± 0.062, *p* < 0.001) compared to controls. Additionally, negative correlations were observed between rCBF in these regions and CDR scores, particularly in the memory domain (rSTG: r = −0.37, *p* = 0.016; right caudate: r = −0.39, *p* = 0.011). **Conclusions:** Reduced rCBF in the rSTG and right caudate may represent early biomarkers for SCD, which could aid in the early detection of AD. These findings suggest that SPECT imaging might be a valuable tool for identifying individuals at risk of cognitive decline, potentially allowing for earlier intervention and targeted preventive strategies in the management of AD.

## 1. Introduction

Alzheimer’s disease (AD) is the most common cause of dementia, an incurable neurodegenerative disorder that accounts for at least two-thirds of dementia cases [1]. Currently, disease-modifying monoclonal antibody therapies for AD aim to promote the clearance of beta-amyloid (Aβ) from the brain. Recent studies have primarily focused on treating patients with amnestic mild cognitive impairment (MCI) or mild AD, where significant neurodegeneration has impaired cognition and the ability to perform the activities of daily living [2,3]. The National Institute on Aging–Alzheimer’s Association (NIA-AA) identifies three stages of AD: dementia due to AD, MCI due to AD, and preclinical (presymptomatic) AD [4]. Several biomarkers exist for the early diagnosis of AD, including amyloid-targeting positron emission tomography (PET) ligands, cerebral hypometabolism in fluorodeoxyglucose PET, and various cerebrospinal fluid (CSF) proteins. However, using these biomarkers to detect preclinical AD in the general population remains challenging due to the high costs of PET imaging, the invasiveness of CSF biomarker collection, and the labor-intensive nature of these procedures [5]. Subjective cognitive decline (SCD) refers to a condition where individuals experience cognitive impairment, such as difficulty remembering names or locating objects while maintaining average performance on standard neuropsychological tests [6]. The prevalence of SCD in the general adult population is relatively high, ranging from 10.4% to 18.8% [7,8]. SCD is considered a potential early indicator of cognitive changes that precede clinically detectable impairment. Although not all individuals with SCD progress to AD, meta-analyses indicate they have approximately double the risk of developing dementia [9]. Longitudinal studies suggest that about 11% of individuals with SCD progress to mild cognitive impairment or dementia over three years [10]. However, the risk of progression varies due to factors like age, APOE ε4 status, and the presence of AD biomarkers [11].

Regional cerebral blood flow (rCBF) abnormalities, as observed through single photon emission computed tomography (SPECT), are well documented in patients with probable AD and MCI [12,13]. Reductions in rCBF have been primarily reported in the bilateral temporoparietal lobes, medial temporal regions, and posterior cingulate cortex, reflecting the severity and progression of both pathological involvement and clinical impairment in AD [14,15,16]. A previous rCBF SPECT study [13] identified reduced cerebral perfusion in the caudal anterior cingulate, posterior cingulate, right insula, adjacent superior temporal gyrus, caudate nucleus, and thalamus in individuals who converted from MCI to AD compared to standard controls. However, there are limited rCBF SPECT data for patients with SCD. A single SPECT study conducted in Japan [17] reported hypoperfusion in the caudate, thalamus, and bilateral temporal regions in patients with SCD. Notably, this study [17] used only the normal standard easy Z-score imaging system (eZIS) as a reference and did not include healthy controls without SCD for comparison.

Moreover, the study by Niwa et al. [17], which investigated rCBF in SCD patients using I-123 IMP SPECT, utilized an insufficient tracer dosage, leading to poor image quality. Although I-123 IMP is considered an effective tracer for cerebral blood flow imaging, administering only half of the recommended dosage significantly reduced the reliability of their findings.

It is important to note that SCD is a heterogeneous concept, encompassing individuals who may progress to dementia as well as those who may not show significant neurodegeneration. As noted by Funaki et al. [18], differentiating between these subgroups remains challenging, even with advanced imaging techniques such as amyloid PET. While amyloid PET scans provide specific insights into amyloid pathology, their limited availability and high costs restrict their routine use in certain clinical and research settings.

Although perfusion changes across the AD spectrum have been extensively studied, less is known about the specific perfusion patterns in SCD. SCD represents a potentially crucial window for early detection and intervention in individuals at risk of cognitive decline. This study aims to address this gap by focusing on subtle perfusion changes that may precede detectable cognitive decline. Specifically, we sought to characterize the distribution of rCBF in patients with SCD compared to age- and sex-matched healthy controls (HCs). We also investigated the correlation between rCBF and various cognitive function domains. We hypothesize that patients with SCD will exhibit reduced rCBF in specific brain regions compared to healthy controls. Furthermore, we expect these reductions to correlate with clinical measures of cognitive function, particularly in memory-related domains. Identifying these perfusion patterns could provide early biomarkers for cognitive decline preceding Alzheimer’s disease.

## 2. Materials and Methods

### 2.1. Study Population

**Figure 1:**
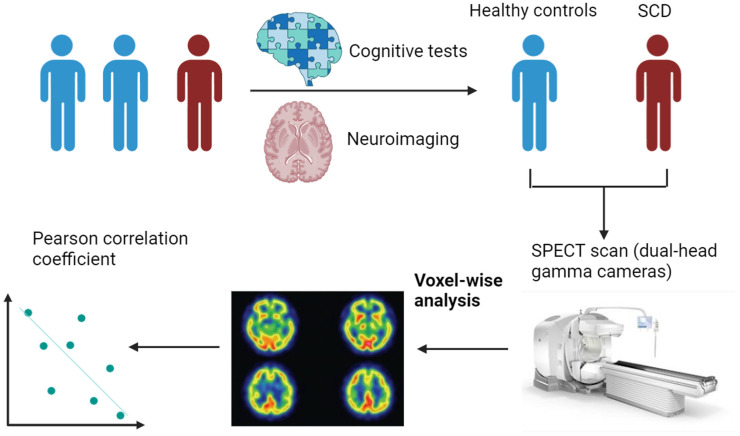
Schematic representation of research methods and procedures. This study was conducted at the memory clinic of Tri-Service General Hospital. After comprehensive cognitive testing and neuroimaging examinations, the study population was divided into two groups (healthy control and SCD). SPECT was used to assess brain function through the measurement of regional cerebral blood flow. Statistical analyses were used to evaluate the correlation between regional cerebral blood flow and cognitive tests in healthy controls and SCD patients. (SCD, subjective cognitive decline; SPECT, single photon emission computed tomography).

Patients aged ≥ 55 years with cognitive-related complaints (i.e., self-reported problems with memory or other cognitive domains) who visited the neurology outpatient department of a tertiary medical center between March 2020 and May 2021 were included for screening if they had undergone both cerebral perfusion imaging and comprehensive neuropsychological tests. The research methodology and procedures are summarized in Figure 1. Cognitive complaints primarily involved memory decline but also included issues in other domains, such as orientation and attention. A diagnosis of SCD was made based on the framework proposed by the SCD Initiative [6], which includes (a) self-reported persistent cognitive complaints, particularly memory decline, within the last five years; (b) persistent concerns about memory changes and feeling inferior to peers of the same age; and (c) performance within normal limits on clinical scales after adjustments for age, sex, and education. To differentiate SCD from other cognitive deficits, we employed a comprehensive neuropsychological battery, including the Mini-Mental State Examination (MMSE), Clinical Dementia Rating (CDR), and Cognitive Abilities Screening Instrument (CASI). Participants were classified as having SCD if they performed within normal limits on all objective tests (MMSE ≥ 27; CASI scores within 1.5 SD of age- and education-adjusted norms) and did not meet the criteria for Mild Cognitive Impairment (MCI) or dementia [19,20,21]. The Geriatric Depression Scale (GDS) was used to exclude participants with severe depression (score > 10) to minimize the impact of affective symptoms on cognitive self-assessment [22].

The exclusion criteria were as follows: patients diagnosed with dementia or MCI; patients diagnosed with Parkinson’s disease or other neurodegenerative diseases; patients with severe cerebrovascular disease (e.g., stroke), high-grade internal carotid artery stenosis (≥50%), brain tumors, or other structural brain diseases; patients with a history of severe head trauma; patients with alcohol or drug abuse; patients with a history of significant or uncontrolled medical illness, such as sepsis, poorly controlled diabetes (hemoglobin A1c > 8.5), heart failure, chronic obstructive pulmonary disease, liver cirrhosis, renal failure, myocardial infarction, or malignancy; and patients with a history of major mental illness that may impair cognitive function, such as major depressive disorder, bipolar disorder, or schizophrenia. Among the 91 patients enrolled for screening, 20 met the criteria for SCD, while another 20 age- and sex-matched HCs were also enrolled for comparison. HCs were required to meet the following requirements: (1) no persistent subjective memory or other cognitive complaints; (2) no evidence of memory or cognitive decline history; (3) no active neurological or mental disorders; (4) no psychotropic drugs; and (5) an MMSE score of >26 for those with middle school education, >22 for primary school education, or >19 for illiterate participants. The demographic and clinical characteristics of the participants are summarized in Table 1. The Institutional Review Board of Tri-Service General Hospital, National Defense Medical Center, Taipei, Taiwan, approved the protocol, and written informed consent was obtained from all participants.

A power analysis was conducted using effect sizes reported in previous studies involving SPECT imaging of cognitive decline [17,18,23]. Based on studies that explored rCBF alterations in patients with SCD and MCI, we determined that a sample size of 20 participants per group would provide 80% power to detect significant differences in rCBF, with an effect size of 0.8 at a significance level of *p* < 0.05.

### 2.2. Neuropsychological Tests

The participants underwent assessments using the MMSE [19], Clinical Dementia Rating (CDR) [24], short-form Geriatric Depression Scale (GDS-S) [25,26], and Cognitive Abilities Screening Instrument (CASI) [20]. The MMSE is a 30-point questionnaire that assesses various cognitive domains, including orientation, memory, language, attention/calculation, and visual construction. It is widely recognized and frequently used as a short screening tool for measuring cognitive impairment in clinical settings [27]. The CDR evaluates six functional categories in patients with dementia: (1) memory; (2) orientation; (3) judgment and problem solving; (4) community affairs; (5) home and hobbies; and (6) personal care. The levels of severity are rated as CDR = 0 (none), CDR = 0.5 (questionable), CDR = 1 (mild), CDR = 2 (moderate), and CDR = 3 (severe). The CDR Sum of Boxes score (CDR-SB) is calculated by summing the scores from the six domains, and it can be used to predict progression and conversion in predementia stages [28]. The GDS-S (Chinese version) is a 15-item questionnaire, with each item contributing 1 point towards the assessment of depression in older adults. Each depressive response scores 1 point, and the total score reflects the number of depressive responses. The scores are categorized as follows: 0–5 (no depression), 6–9 (suggestive of mild depression), and ≥10 (suggestive of severe depression). The CASI score ranges from 0 to 100, and the total score is derived from nine cognitive domains, including long-term memory, short-term memory, attention, mental manipulation and concentration, orientation, abstract thinking and judgment, language, visual construction, and list-generating fluency.

### 2.3. Cerebral Perfusion Imaging Acquisition and Analysis

#### 2.3.1. Cerebral Perfusion SPECT Acquisition

Cerebral perfusion imaging was performed using dual-head gamma cameras (Discovery NM/CT 670 and Discovery NM/CT 870DR; GE Healthcare, Waukesha, WI, USA) equipped with low-energy fan beam collimators. All participants received intravenous injections of a single bolus dose of 740 MBq (20 mCi) ^99m^Tc-ethyl cysteine dimer (^99m^Tc-ECD). Following injection, patients were placed in a quiet, dimly lit room for 30 min to allow radiotracer uptake. SPECT images were acquired after the uptake period using a standardized imaging protocol. Patients were positioned supine in a low-light environment with minimal noise to minimize external stimuli. The acquisition parameters for SPECT were as follows: matrix size, 128 × 128; voxel size, 4.42 × 4.42 × 4.42 mm^3^; 60 frames (45 s per frame); and a 10% symmetric energy window at 140 keV. Images were reconstructed using filtered back projection with a Butterworth filter (cut-off frequency, 0.45 cycles/pixel; power, 15). Uniform Chang attenuation correction was applied to compensate for photon attenuation. The final reconstructed image had a matrix size of 128 × 128 × 32 and a voxel size of 2.25 × 2.25 × 4.5 mm^3^.

#### 2.3.2. Image Pre-Processing

All image data were pre-processed using PMOD image analysis software (version 3.8; PMOD Technologies Ltd., Zurich, Switzerland). The SPECT images were spatially normalized to the template [29]. Volumes of interest in the entire cerebellum were selected based on the automated anatomic labeling atlas [30]. Uptake ratio (UR) images for voxel-wise analysis were obtained by dividing each voxel’s uptake by the cerebellum’s mean uptake [31].

#### 2.3.3. Voxel-Wise Analysis

Voxel-wise analysis was performed using Statistical Parametric Mapping 12 (SPM12). A voxel-wise two-sample *t*-test was employed to compare UR images between patients with SCD and HCs. Age, sex, education, and GDS score were included as covariates to control for the potential influence of these factors on UR images. The adjustment of rCBF values for age, sex, and GDS score was necessary to account for known confounders that can impact cerebral blood flow patterns. Age-related decreases in cerebral blood flow (CBF) [32], sex-based differences in CBF patterns [33], the influence of educational attainment on cognitive reserve and CBF [34], and the effects of depressive symptoms on CBF [35] are well documented. By adjusting for these variables, we aimed to isolate the effects of SCD on rCBF patterns more accurately. To address the problem of multiple comparisons in image-based analyses, we applied the AlphaSim command-line tool, available in the AFNI toolbox on Ubuntu Linux version 20.04 (Analysis of Functional NeuroImages, http://afni.nimh.nih.gov/afni/) (accessed on 20 August 2024). The statistical threshold for each voxel was set at corrected *p*_alpha_ < 0.05, with a minimum cluster size of 297 voxels, as determined by the Monte Carlo simulation. The mean volume of each significant cluster was extracted for each participant and correlated with the clinical evaluations. Coordinates of the voxels within significant clusters were transformed from Montreal Neurological Institute (MNI) space to Talairach coordinates using the GingerALE toolbox version 3.0.2 (The BrainMap Development Team; http://brainmap.org/ale/index.html) (accessed on 20 August 2024). The anatomical structures of these significant clusters were identified using the Talairach and Tournoux atlas [36].

### 2.4. Statistical Analysis

Quantitative variables were reported as mean ± standard deviation. We used the Student’s *t*-test to compare the patients with SCD and HCs. Associations between URs and clinical characteristics were assessed using Pearson’s correlation analysis. A *p*-value of 0.05 or less was considered significant. All statistical analyses were performed using the Statistical Package for Social Sciences (SPSS version 20 for Windows^®^, SPSS Inc., Chicago, IL, USA).

## 3. Results

### 3.1. Demographic and Clinical Data

The demographic variables of the participants are summarized in Table 1. The HC and SCD groups were comparable in terms of mean age, sex distribution, and years of education (age: 71.00 ± 6.66 vs. 71.35 ± 6.68, *p* = 0.87; sex ratio [M/F]: 9/11 vs. 8/12, *p* = 0.76; years of education: 11.25 ± 4.51 vs. 11.60 ± 3.97, *p* = 0.80). The mean MMSE scores did not differ significantly between the two groups (27.80 ± 1.96 vs. 28.45 ± 0.94, *p* = 0.19), and the mean CASI scores were also similar (90.60 ± 7.55 vs. 92.73 ± 3.55, *p* = 0.26). However, the “language” domain scores of the HCs were slightly lower than those in the SCD group (9.31 ± 1.19 vs. 9.91 ± 0.25, *p* = 0.03). The mean Global CDR score of patients with SCD was slightly higher than that of HCs (0.33 ± 0.24 vs. 0.50 ± 0.00, *p* = 0.003), with notable differences observed mainly in the memory domain (0.35 ± 0.29 vs. 0.50 ± 0.00, *p* = 0.024).

### 3.2. Voxel-Wise Comparisons of Cerebral Perfusion Imaging in Patients with SCD and HCs

**Figure 2:**
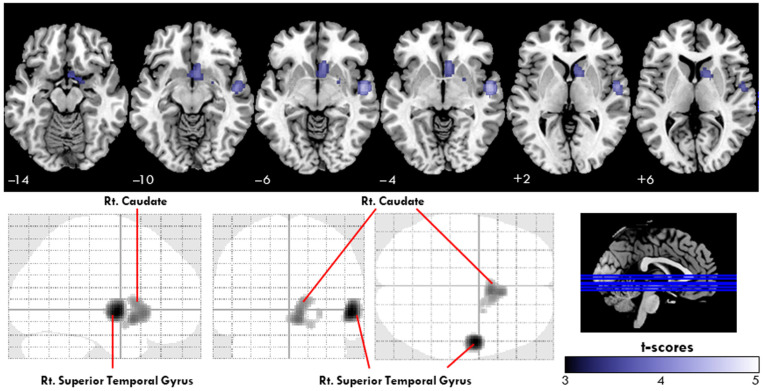
Regions showing significant differences in uptake ratio (UR) between patients with subjective cognitive decline (SCD) and healthy controls (HCs), as revealed by voxel-based analysis. The cold color map showed a significant reduction in URs in the right superior temporal gyrus and right caudate in patients with SCD. Glass-brain images showed the spatial distribution of UR reduction in patients with SCD (corrected *p* < 0.05). No area of increased UR was found in the SCD group compared to the HC group. (SCD, subjective cognitive decline; Rt, right).

Figure 2 illustrates the differences in cerebral perfusion between patients with SCD and HCs, with the cluster-level statistics for the significantly different regions summarized in Table 2. Regional cerebral blood flow (rCBF) in the right superior temporal gyrus (rSTG) was significantly lower in the SCD group compared to the healthy controls. Specifically, the mean uptake ratio in the rSTG was 0.864 ± 0.090 for the SCD group, compared to 1.030 ± 0.074 for the control group (*p* < 0.001, 95% confidence interval (CI): −0.1264 to −0.03918). Similarly, the mean uptake ratio in the right caudate was 0.783 ± 0.068 for the SCD group, compared to 1.030 ± 0.074 for the control group (*p* < 0.001, 95% CI: −0.1327 to −0.03058).

### 3.3. Relationship Between Regional UR Changes and Clinical Variables

We examined the correlations between regional uptake ratios (rURs) in the rSTG and right caudate with various cognitive measures (Table 3, Figure S1). The analysis of these correlations included data from all study participants—both the SCD group and the healthy control group. Significant negative correlations were observed between rURs in both regions and CDR scores. Specifically, rSTG rUR was significantly negatively correlated with Global CDR (r = −0.36, *p* = 0.019), CDR Memory subscale (r = −0.37, *p* = 0.016), and CDR Sum of Boxes (CDR-SB) (r = −0.31, *p* = 0.046). Similarly, right caudate rUR demonstrated significant negative correlations with Global CDR (r = −0.36, *p* = 0.021), CDR Memory subscale (r = −0.39, *p* = 0.011), and CDR-SB (r = −0.35, *p* = 0.024). Most of the Cognitive Abilities Screening Instrument (CASI) subscales did not show significant correlations with rURs, except for a positive correlation between right caudate rUR and long-term memory (r = 0.34, *p* = 0.027).

**Figure 3:**
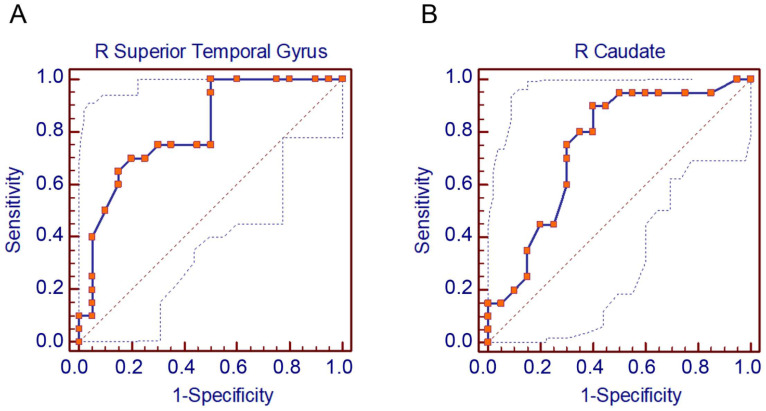
(**A**) Receiver operating characteristic (ROC) curves are shown for the right superior temporal gyrus. The area under the curve (AUC) is 0.810. (**B**) Receiver operating characteristic (ROC) curves are shown for the right caudate nucleus. The area under the curve (AUC) is 0.746. (R = Right).

The results of the receiver operating characteristic (ROC) curve analysis are shown in Figure 3A,B. The data indicated that the rSTG had excellent discriminatory power for predicting patients with SCD, whereas the right caudate nucleus demonstrated acceptable power. The areas under the ROC curve (AUC) for the rSTG and right caudate nucleus were 0.810 and 0.746, respectively. For the rSTG, the AUC was 0.810, with a standard error of 0.0694 (95% confidence interval [CI]: 0.655 to 0.916; z statistic: 4.469; significance level [area = 0.5]: *p* < 0.0001; Youden index J value: 0.5000; associated criterion: ≤0.95). For the right caudate nucleus, the AUC was 0.746, with a standard error of 0.0815 (95% CI: 0.584 to 0.870; z statistic: 3.020; significance level [area = 0.5]: *p* = 0.0025; Youden index J value: 0.5000; associated criterion: ≤0.84). The corrected optimal cut-off based on the ROC curve for the rSTG was 0.95 (UR), with a sensitivity of 65.0% and a specificity of 85.0% (Figure 3A). The corrected optimal cut-off based on the ROC curve for the right caudate nucleus was 0.84 (UR), with a sensitivity of 90.0% and a specificity of 60.0% (Figure 3B).

## 4. Discussion

The present case-control study investigated the characteristics of rCBF in patients with SCD. The rCBF was lower in the rSTG and right caudate in the SCD group than in HCs. Moreover, significant negative correlations were observed between rCBF in the rSTG and right caudate and CDR scores, particularly in the memory domain.

In a previous rCBF SPECT study conducted in Japan, patients with subjective cognitive impairment showed significant hypoperfusion in both temporal areas, the caudate, and the thalamus compared to the eZIS standard database [17]. Subjective cognitive impairment may be an early indicator of cognitive decline. However, the relationship between SCD and cognitive function is likely influenced by various confounding factors, including age, sex, education level, and comorbidities such as depression [37]. Notably, the previous study [17] did not enroll a healthy control group, which precluded adjustment for these possible confounders. Additionally, subjective cognitive impairment was not defined according to consensus criteria in that study. In designing the present study, we compared individuals with SCD, defined according to the SCD Initiative [6], with an age- and sex-matched healthy control group to explore the detailed characteristics of rCBF distribution while controlling for confounding factors such as age, sex, education level, and depressive symptoms. Our SPECT study demonstrated that, compared to HCs, patients with SCD had significantly lower uptake in the rSTG and right caudate.

### 4.1. Correlations Between Memory Function and rCBF

In our study, we found significant negative correlations between reduced URs in the rSTG and right caudate and CDR scores, particularly in the memory domain. Specifically, lower rCBF in these regions was associated with higher (worse) Global CDR, CDR Memory subscale, and CDR Sum of Boxes (CDR-SB) scores. These findings suggest a potential relationship between reduced perfusion in these regions and early cognitive changes in SCD. Interestingly, most Cognitive Abilities Screening Instrument (CASI) subscales did not show significant correlations with rURs, except for a positive correlation between right caudate UR and long-term memory. This discrepancy between CDR and CASI correlations may indicate that rCBF changes in the rSTG and right caudate are more sensitive to the subtle cognitive changes captured by the CDR in the SCD stage rather than the more specific cognitive domains assessed by the CASI.

These findings may suggest that individuals with SCD, at an earlier stage on the continuum of cognitive decline (a preclinical asymptomatic stage of AD), may still be able to compensate for cognitive challenges in specific domains while showing subtle changes detectable by more global measures like the CDR. More longitudinal studies evaluating patients with SCD, MCI, and AD using the same methods are warranted to investigate these possibilities. Notably, the mean Global CDR score, CDR Memory subscale score, and CDR-SB for the SCD group were 0.5 in our study, similar to the findings of a previous study conducted in Taiwan [38]. This consistency supports the reliability of our CDR assessments in characterizing the SCD group.

### 4.2. Superior Temporal Gyrus and Cognitive Functions

The superior temporal gyrus (STG) is located inferior to the lateral sulcus and superior to the superior temporal sulcus. It is responsible for auditory word comprehension, speech perception, and the processing of non-verbal social cues [39]. In our study, we found that the rSTG UR was reduced in patients with SCD compared to HCs. Similar to our findings, a previous meta-analysis of structural magnetic resonance imaging studies suggested that patients with major depressive disorder (MDD) and MCI shared volumetric reductions in many regions, including the insula, STG, inferior frontal gyrus, amygdala, thalamus, and hippocampus [40]. Among these, the STG is crucial, as it is part of a language network, while the insula contributes to cognitive functions, auditory processing, and socio-emotional processing [41]. The authors proposed that volume reductions in the STG and insula reflect deficits in communication and reduced participation in socially, mentally, or cognitively stimulating activities—known risk factors for MCI and MDD [40]. The STG can be divided into anterior, middle, and posterior segments. The temporal–parietal junction, which includes the ventral portions of the parietal cortex and the posterior segment of the STG, is involved in cognitive functions such as social cognition, episodic memory retrieval, and bottom-up attention [42].

### 4.3. Caudate and Cognitive Functions

The caudate has been implicated in cognitive functions, including memory and learning [43]. The pattern and degree of hypoperfusion we observed in SCD differ from those seen in other neurodegenerative conditions. For instance, in AD, caudate hypoperfusion is typically less pronounced than in regions such as the posterior cingulate and temporoparietal cortex [23]. The role of the caudate in early cognitive decline, particularly in SCD, is not well established. Some studies have suggested that caudate volume and function may be associated with cognitive performance in aging and mild cognitive impairment (MCI) [44]. However, the specific implications of caudate hypoperfusion in SCD remain to be elucidated. It is important to note that the degree of hypoperfusion observed in SCD is subtle compared to the severe hypometabolism seen in some neurodegenerative disorders. This finding suggests that rCBF SPECT may be helpful in detecting early changes in cerebral perfusion that precede more severe cognitive decline.

The cluster we identified in the caudate region may partially overlap with the orbitofrontal cortex, a finding that warrants further discussion. Iizuka and Kameyama [45] demonstrated that the right orbitofrontal region is involved in the awareness of dementia with Lewy bodies, while Perrotin et al. [46] showed its involvement in anosognosia in AD. This potential overlap could suggest that the reduced rCBF we observed might be related to alterations in self-awareness or metamemory processes, which are crucial aspects of subjective cognitive decline (SCD). The orbitofrontal cortex has been implicated in self-referential processing and the integration of sensory information with emotional and motivational states [47]. Therefore, the reduced perfusion in this region could potentially explain the discrepancy between the subjective complaints and objective cognitive performance that characterizes SCD.

However, it is important to note that our study design did not specifically investigate the orbitofrontal cortex, and the spatial resolution of SPECT imaging makes it challenging to distinguish between caudate and nearby orbitofrontal activations. Future studies using higher-resolution imaging techniques, such as high-resolution PET or 7T fMRI, could more precisely localize these activation patterns and clarify their relationship to SCD symptoms.

MRI offers advantages such as non-invasive assessment and concurrent structural imaging during arterial spin labeling. We selected SPECT for this study due to its established sensitivity in detecting subtle perfusion changes in SCD [48], superior quantification accuracy in low-flow states, and immediate clinical translatability. SPECT’s ability to provide absolute quantification of cerebral blood flow is valuable for studying early-stage changes in SCD. However, the use of Tc-99m ECD as our SPECT tracer presents certain limitations. As Kameyama [49] noted, its low permeability–surface area product leads to a reduced contrast across brain regions. This limitation may have affected our ability to identify significant differences in other important regions beyond the rSTG and right caudate, potentially masking subtle but clinically relevant perfusion changes in areas implicated in early cognitive decline. Future studies might consider using tracers with higher permeability–surface area products to detect more subtle and widespread changes in cerebral blood flow patterns.

The strengths of our study include the robust statistical analysis, detailed cognitive examinations, and the use of a standard research diagnostic tool provided by the SCD Initiative [6]. Nevertheless, several limitations of the present study require consideration. First, a primary limitation is the modest sample size. Therefore, researchers should view the findings of this study as preliminary and validate them in larger, independent cohorts. Second, our study’s inability to distinguish between SCD individuals who may progress to dementia and those who may not is a significant limitation. Without CSF biomarkers or amyloid PET data, we cannot definitively categorize our SCD group in terms of AD pathology or the likelihood of progression. This limitation is common in SCD research, as highlighted by Funaki et al. [18], who found that differentiating SCD subgroups remains challenging due to small sample sizes. Furthermore, we acknowledge that our findings of reduced rCBF in the rSTG and right caudate, rather than the posterior cingulate, differ from what might be expected in early AD. As noted by Minoshima et al. [50] and Kumakura et al. [51], the posterior cingulate often shows the earliest decrease in blood flow in AD. Our differing results could be due to the heterogeneity of our SCD group, the specific characteristics of our sample, or methodological differences. This finding underscores the need for further research to clarify the relationship between SCD, rCBF patterns, and progression to AD. Finally, the absence of AD or MCI groups for comparison is another limitation. Future research should include these groups to provide a more comprehensive understanding of the continuum of rCBF changes from SCD through MCI to AD. This addition would clarify the specificity and sensitivity of the observed rCBF reductions as potential biomarkers for early AD-related symptoms [52,53]. Such an expanded study design could also help determine whether the rCBF patterns we observed in SCD are predictive of progression to MCI or AD.

Our findings of reduced rCBF in the rSTG and right caudate in SCD patients have significant implications for early AD detection. These rCBF changes may serve as early biomarkers of cognitive decline, preceding detectable cognitive impairment and reinforcing the neurobiological basis of SCD [52,53]. This aligns with and potentially refines the current AD continuum model, suggesting that perfusion changes may occur earlier than previously thought [5]. These results could impact clinical trials by providing new inclusion criteria or outcome measures for early-stage interventions [54]. Moreover, the correlation between rCBF reductions and CDR scores suggests the potential for personalized risk assessment [55]. However, our study has limitations, including its cross-sectional design, modest sample size, and the lack of AD-specific biomarkers. Future research should address these gaps through longitudinal studies with larger, more diverse samples, the incorporation of amyloid and tau biomarkers, and the inclusion of MCI and early AD groups for comparison. Such studies will be crucial for validating our findings and potentially improving early diagnosis and management strategies for individuals at risk of cognitive decline.

## 5. Conclusions

Our study identifies significant reductions in regional cerebral blood flow (rCBF) in the right superior temporal gyrus (rSTG) and right caudate in patients with subjective cognitive decline (SCD). These findings suggest that these regions could serve as early biomarkers for cognitive decline, preceding more overt neurodegenerative changes typical of Alzheimer’s disease. By identifying perfusion deficits in SCD patients, this research contributes to the growing body of evidence that functional changes in the brain occur prior to structural degeneration in the Alzheimer’s continuum. Furthermore, the potential use of rCBF changes in the rSTG and right caudate as early indicators of cognitive decline has significant implications for clinical practice. Non-invasive imaging tools like SPECT could be integrated into routine screening processes to identify individuals at a higher risk of progression to Alzheimer’s disease, enabling earlier intervention strategies aimed at mitigating or delaying disease progression. Future research should focus on longitudinal studies to validate these findings and explore interventions that could target these specific brain regions to slow cognitive decline in at-risk populations.

## Figures and Tables

**Table 1:** Demographic and clinical characteristics of the HC and SCD groups.

Demographic Variable	Healthy Control	SCD Group	*p*
Age (yr)	71.00 ± 6.66	71.35 ± 6.68	0.869
Sex (M/F)	9/11	8/12	0.757
Education (year)	11.25 ± 4.51	11.60 ± 3.97	0.796
GDS score	3.40 ± 2.56	4.00 ± 2.47	0.456
MMSE score	27.80 ± 1.96	28.45 ± 0.94	0.190
CASI score	90.60 ± 7.55	92.73 ± 3.55	0.259
Long-term memory	9.85 ± 0.49	9.85 ± 0.49	1.000
Short-term memory	10.79 ± 0.97	11.27 ± 0.90	0.108
Attention	7.30 ± 0.92	7.75 ± 0.55	0.069
Mental manipulation	8.95 ± 1.54	9.25 ± 1.02	0.472
Orientation	17.15 ± 1.76	17.15 ± 1.23	1.000
Abstraction and judgement	9.75 ± 1.25	9.90 ± 1.12	0.692
Language	9.31 ± 1.19	9.91 ± 0.25	0.033
Visual construction	9.75 ± 1.12	9.80 ± 0.62	0.862
List-generating fluency	7.75 ± 2.17	7.90 ± 1.89	0.817
Global CDR	0.33 ± 0.24	0.50 ± 0.00	0.003
CDR-SB	0.40 ± 0.45	0.50 ± 0.00	0.324
CDR Memory domain	0.35 ± 0.29	0.50 ± 0.00	0.024

Data are presented as mean ± standard deviation. HC, healthy control; SCD, subjective cognitive decline; GDS, Geriatric Depression Scale; MMSE, Mini-Mental State Examination; CASI, Cognitive Abilities Screening Instrument; CDR, Clinical Dementia Rating; CDR-SB, Clinical Dementia Rating Sum of Boxes score.

**Table 2:** Anatomical regions showing significant reductions in the uptake ratio (UR) in the patients with SCD compared with those in the healthy control group.

MNI Coordinates			Voxel Size	Left or Right	Anatomical Region	Brodmann Area	Regional Uptake Ratio, Mean ± SD		T Score ^a^
x	y	z					Healthy Control	SCD Group	
54	−4	−4	440	Right	Superior Temporal Gyrus	22	1.030 ± 0.074	0.864 ± 0.090	4.48
6	10	−10	536	Right	Caudate	Caudate Head	0.947 ± 0.062	0.783 ± 0.068	3.95
8	18	−6		Right	Caudate	Caudate Head			3.89
10	12	2		Right	Caudate	Caudate Body			3.75

MNI, Montreal Neurological Institute; SD, standard deviation. ^a^ T scores are for each regional cluster’s peak statistically significant voxel with corrected *p* < 0.05 (corrected using a Monte Carlo simulation), controlled for age, sex, Geriatric Depression Scale score, and education.

**Table 3:** Pearson correlations between regional uptake ratios (rSTG and right Caudate) and clinical parameters.

Anatomical Region	CASI	Long-Term Memory	Short-Term Memory	Attention	Mental Manipulation	Orientation	Abstraction and Judgement	Language	Visual Construction	List-Generating Fluency	Global CDR	CDR-SB	CDR M
R Superior Temporal Gyrus	0.08 (0.595)	0.26 (0.103)	−0.08 (0.591)	0.07 (0.652)	0.16 (0.316)	−0.04 (0.784)	0.17 (0.285)	0.01 (0.932)	0.11 (0.487)	−0.03 (0.829)	−0.36 (0.019) *	−0.37 (0.016) *	−0.31 (0.046) *
R Caudate	0.16 (0.310)	0.34 (0.027) *	0.04 (0.804)	0.13 (0.410)	0.24 (0.134)	−0.02 (0.863)	0.11 (0.492)	0.08 (0.582)	0.18 (0.256)	0.003 (0.981)	−0.36 (0.021) *	−0.39 (0.011) *	−0.35 (0.024) *

Values represent correlation coefficients with *p*-values in parentheses. Asterisks denote statistically significant correlations (*p* < 0.05).

## Data Availability

The datasets obtained and analyzed during the current study are available from the corresponding author upon reasonable request.

## Supplementary Materials

The following supporting information can be downloaded at: https://www.mdpi.com/article/10.3390/jcm13226855/s1, Figure S1: Scatter plots showing correlations between regional uptake ratios in the right superior temporal gyrus (A) and right Caudate (B) with various cognitive measures from CASI (A), CASI subdomains (B–J), Global CDR (K), CDR M domain (L) and CDR-SB (M). Blue dots represent healthy controls, and red dots represent the SCD group. Pearson correlation coefficients (r) are displayed for each plot, with asterisks denoting statistically significant correlations *(p* < 0.05).
