# Supplementary material for: Characterizing Cerebral Perfusion Changes in Subjective Cognitive Decline Using Single Photon Emission Computed Tomography: A Case-Control Study
Source: J Clin Med. 2024 Nov 14;13(22):6855. doi: 10.3390/jcm13226855 (PMC11595019; doi:10.3390/jcm13226855)
Supplement: Supplementary file 1 [file jcm-13-06855-s001.zip › jcm-3247333-supplementary.pdf]

Supplementary Materials

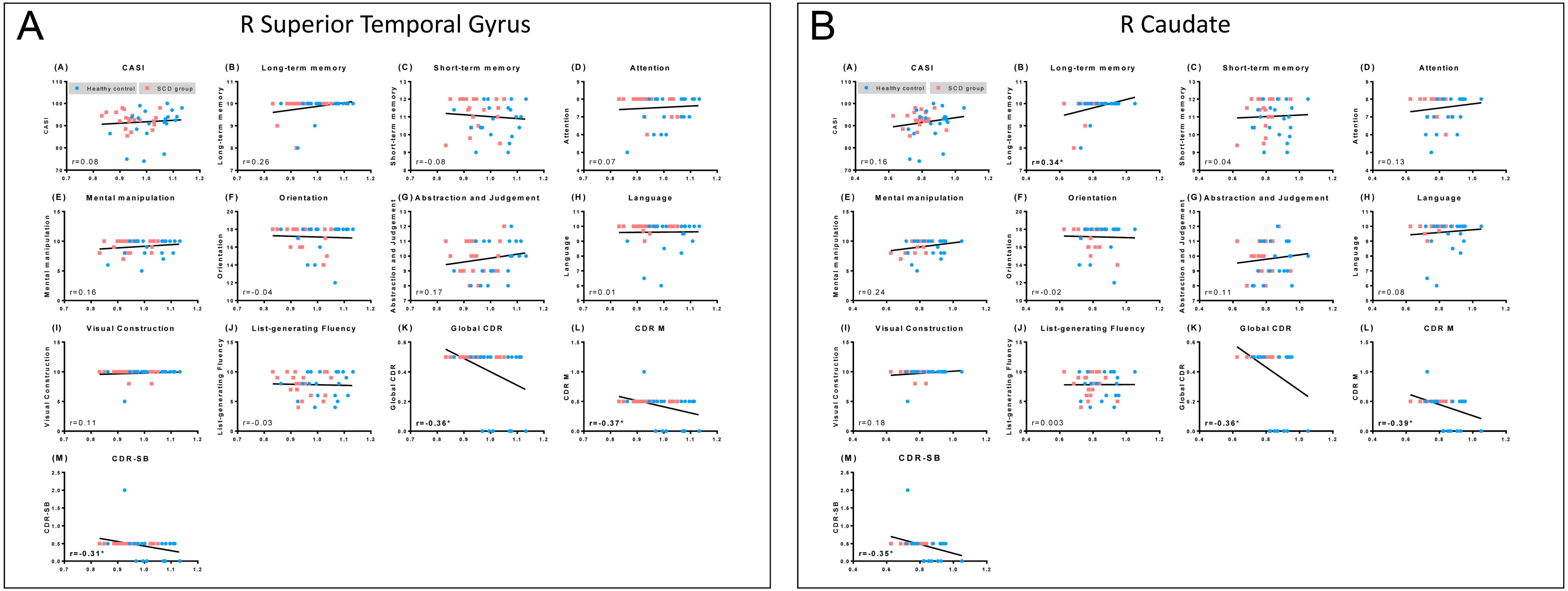

Figure S1. Scatter plots showing correlations between regional uptake ratios in the right superior temporal gyrus (**A**) and right Caudate (**B**) with various cognitive measures from CASI (**A**), CASI subdomains (**B**–**J**), Global CDR (**K**), CDR M domain (**L**) and CDR-SB (**M**). Blue dots represent healthy controls, and red dots represent the SCD group. Pearson correlation coefficients (r) are displayed for each plot, with asterisks denoting statistically significant correlations ($p < 0.05$).
